# Human iPSC-derived glia models for the study of neuroinflammation

**DOI:** 10.1186/s12974-023-02919-2

**Published:** 2023-10-10

**Authors:** Nina Stöberl, Emily Maguire, Elisa Salis, Bethany Shaw, Hazel Hall-Roberts

**Affiliations:** UK Dementia Research Institute (UK DRI), School of Medicine, Cardiff University, Cardiff, CF10 3AT UK

**Keywords:** Induced pluripotent stem cells, iPSC, Neuroinflammation, Microglia, Astrocytes, Monoculture, Co-culture, Neural organoids, Xenotransplantation, Cytokines

## Abstract

Neuroinflammation is a complex biological process that plays a significant role in various brain disorders. Microglia and astrocytes are the key cell types involved in inflammatory responses in the central nervous system. Neuroinflammation results in increased levels of secreted inflammatory factors, such as cytokines, chemokines, and reactive oxygen species. To model neuroinflammation in vitro, various human induced pluripotent stem cell (iPSC)-based models have been utilized, including monocultures, transfer of conditioned media between cell types, co-culturing multiple cell types, neural organoids, and xenotransplantation of cells into the mouse brain. To induce neuroinflammatory responses in vitro, several stimuli have been established that can induce responses in either microglia, astrocytes, or both. Here, we describe and critically evaluate the different types of iPSC models that can be used to study neuroinflammation and highlight how neuroinflammation has been induced and measured in these cultures.

## Background

### Defining neuroinflammation

Neuroinflammation is commonly used to describe pathology in multiple central nervous system (CNS) conditions, including Alzheimer’s disease (AD), Parkinson’s disease (PD), multiple sclerosis (MS), and amyotrophic lateral sclerosis (ALS) [1]. Unfortunately, definitions of what constitutes neuroinflammation vary widely across the literature. Within the CNS, the inflammatory response is primarily driven by glia cells, namely microglia and astrocytes [1, 2]. These cells mediate the response by releasing cytokines, chemokines, reactive oxygen species (ROS), and secondary messengers. Cytokines can be pro- or anti-inflammatory, thereby either exacerbating or dampening the immune response. For the purpose of this review, we define neuroinflammation as the presence of inflammatory mediators within the CNS [3].

Many researchers have previously classified microglia and astrocytes into dichotomous ‘good’ versus ‘bad’ states (e.g. M1 & M2 microglia, A1 & A2 astrocytes) [4, 5]. Traditionally, cells representing M1 and A1 states were assumed to serve detrimental roles within diseases, propagating uncontrolled, damaging, inflammatory responses. These M1 and A1 cells have often been referred to as 'activated’ (microglia) or ‘reactive’ (astrocytes), thus distinguishing them from the ‘resting’ cells found in an unstimulated brain. In contrast, M2 and A2 cells were considered ‘protective’ within diseased brains, acting to limit inflammatory responses and initiate tissue repair processes. Recent research, however, has proved these rigid, binary definitions to be largely unfounded [6, 7]. Rather than adhering to such strict categories, the states of these glia cells are highly dynamic, and they exhibit complex, fluid responses to varied stimuli and environments [6, 7].

Microglia and astrocytes do not initiate inflammatory responses independently. Instead, they engage in communication with each other as well as with other types of cells in the CNS, including neurons [8]. In general, upon detection of an insult, the secretion of inflammatory factors by microglia (e.g. IL-1α, TNF and C1q) stimulates astrocytes to acquire a more reactive, inflammatory phenotype [9]. In turn, reactive astrocytes likely secrete additional factors that affect microglia-mediated neuroinflammatory behaviours [10]. Depending on the stimuli, distinct activation modes are initiated in these immune cells, although often involving shared signalling pathways and effectors [11]. During CNS injury and disease, microglia and astrocytes can serve both protective and detrimental roles depending on the specific context [12]. Further details about inflammatory signalling pathways and cytokine functions in general, as well as for the pathogenesis of Alzheimer’s disease, have already been described elsewhere [13, 14].

### Microglia

Microglia are considered the main immune cells of the CNS. These cells make up between 0.5 and 16.6% of the total cell population in the human brain, depending on anatomical region, sex, and stage of development, amongst other variables [15, 16]. Microglia possess multiple branched processes, or ramifications, to survey the CNS environment [17]. Despite previous assumptions that microglia were quiescent within the healthy adult brain, they are now known to be crucial players both during development and in maintaining normal brain homeostasis [18]. In brief, the main functions of microglia are to mediate the inflammatory response by secreting cytokines and chemokines, and to phagocytose unwanted material and synapses [19]. Microglia are highly versatile both functionally and morphologically and can rapidly adapt in response to a diverse range of stimuli [20].

Upon detection of either pathogen-associated molecular patterns (PAMPs), damage-associated molecular patterns (DAMPs), or neurodegeneration-associated molecular patterns (NAMPs), microglia undergo a rapid phenotypic change [21, 22]. NF-κB (nuclear factor κ-light-chain-enhancer of activated B cells), a pleiotropic regulator of many cellular signalling pathways, plays a major role in facilitating the neuroinflammatory response of not only microglia, but also astrocytes, to these stimuli [23]. Activated microglia migrate to the site of damage and injury via a process called chemotaxis, as well as release chemokines (e.g. CCL2 and CXCL1), pro- and anti- inflammatory cytokines (e.g. IL-12, TNF, IL-10, TGF-β), and a variety of other inflammatory mediators [21]. These mediators then further stimulate immune responses in other glia cells [24]. Under certain disease conditions, microglia can also be seen to proliferate and undergo morphological changes (often taking on a more amoeboid appearance). This response, termed microgliosis, likely increases the ability of these cells to survey the brain parenchyma and to migrate more easily towards insults [20]. Transcriptomic studies have revealed the ability of microglia to acquire a vast diversity of transcriptomic states in response to different disease conditions. This includes forming a population of ‘disease-associated microglia’, which have increased gene expression involved with phagocytosis and lipid metabolism [25]. While there remains difficulty in directly correlating these different transcriptional states with differences in cell function, transcriptomics can provide insight into the heterogeneity of glia in neuroinflammatory conditions.

### Astrocytes

Astrocytes act as the most prevalent form of glia, making up 17–61% of the total cell numbers in the CNS [26]. These highly heterogeneous cells display a variety of densities, morphologies, gene expression, and proliferation rates depending on many factors including brain region and disease state [27]. Astrocytes have roles in innate and adaptive immunity, neurogenesis, providing metabolic support to neurons, maintaining blood–brain barrier integrity, and are implicated in learning and memory [27, 28]. Astrocytes form intimate connections with neurons, thus allowing them to sensitively detect neuronal damage and effectively regulate the subsequent inflammatory response. Astrocytes remove neuronal-secreted glutamate from the synaptic cleft, thus reducing glutamate-induced excitotoxicity and inflammation [29]. Furthermore, dying neurons release ATP and potassium, both of which can induce inflammasome activation within astrocytes, which in turn leads to the release of pro-inflammatory chemokines and cytokines [30, 31]. In general, the role of astrocytes following detection of harmful stimuli is to regulate the resulting inflammation and push the brain environment towards a more homeostatic state [32]. However, under certain conditions, astrocytes can also be seen to contribute to both neuroinflammation and tissue damage [33].

Within many disease states, astrocytes have been described as taking on a more ‘reactive’ phenotype, characterized by increased expression of the glial fibrillary acid protein (GFAP) [7]. This reactive phenotype involves cell proliferation and hypertrophy (also known as astrogliosis), with astrocytes clustering and integrating with extracellular matrix components to form a ‘glial scar’. The glial scar physically shields the injured region from neighbouring healthy tissue, thus preventing the spread of inflammatory mediators and debris [34]. In addition, ‘reactive’ astrocytes activate NF-κB and produce a variety of chemokines (e.g. CCL2, CXC3L1, CXCL1) that attract other immune cells and cytokines (e.g. IFN-γ, IL-12, TNF, IL-10, and TGF-β), which propagate the immune response by stimulating neighbouring glia [35, 36].

### Induced pluripotent stem cells (iPSC)

**Fig. 1:**
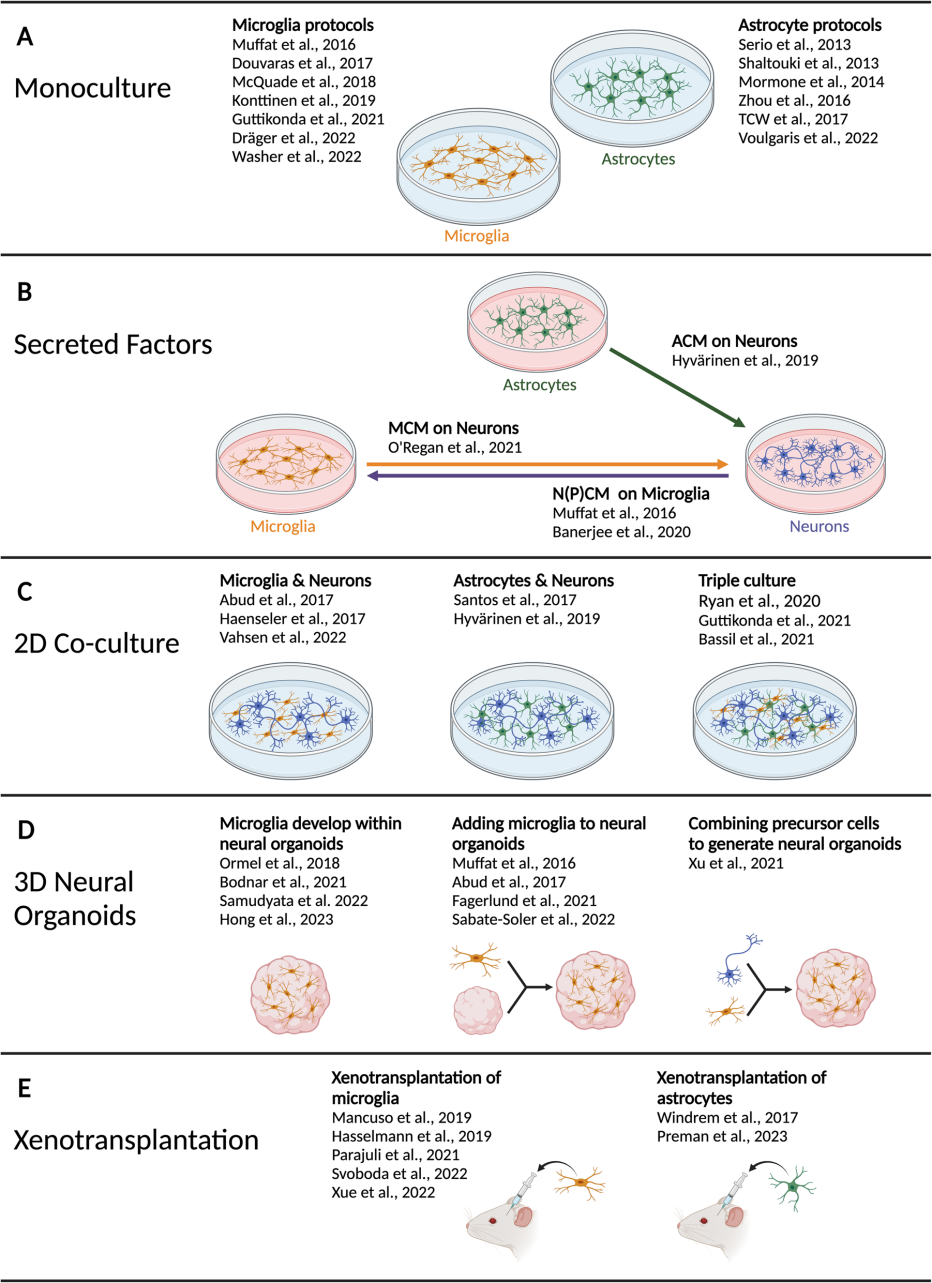
Overview of culture models for iPSC-microglia and iPSC-astrocytes. **A** In recent years, numerous protocols have been developed to differentiate iPSC to microglia and astrocytes, of which a selected number is mentioned here. **B** PSC-derived cell conditioned media has been used to investigate its effect on other CNS cell types. Here we list all the studies mentioned in the review. **C** iPSC-derived CNS cells can also be studied in co-cultures of two or three different cell types. **D** Several strategies for the generation of 3D neural organoids have been established and a number of representative protocols are highlighted here. **E** Recently, methods to transplant human pluripotent stem cell-derived microglia and astrocytes into rodent brains have been established. The lists of protocols mentioned in this figure are not exhaustive

iPSC, initially generated in 2006 by Takahashi and Yamanaka [37], have enormous potential in a wide variety of medical and research applications, as is reviewed extensively elsewhere [38]. These artificial stem cells are formed from somatic cells (e.g. fibroblastsor blood) following the overexpression of transcription factors that stimulate de-differentiation to a state similar to embryonic stem cells. Once formed, these iPSC are capable of infinite self-renewal and can differentiate into all three embryonic germ layers, and more specialized cell types. Protocols have been developed for the differentiation of iPSC into cells of the CNS, including neurons, microglia, and astrocytes, and have been reviewed elsewhere [39–41]. These cells can then be studied in monoculture (single cell type), 2D co-culture (multiple cell types), or in 3D culture (e.g. neural organoids). In addition, iPSC-derived cells can be studied post-injection into a mouse brain (xenotransplantation) [42] (Fig. 1).

iPSC-derived microglia and astrocytes can produce inflammatory responses that share many similarities with corresponding in vivo responses [43, 44]. This includes secretion of cytokines, chemokines, ROS, and other inflammatory mediators. The neuroinflammatory response in vivo involves numerous complex interactions between multiple brain cell types. For this reason, inflammatory responses are likely to be more physiologically relevant with increasing culture complexity.

### Models for studying neuroinflammation

A great number of models to study neuroinflammatory responses are available, ranging from immortalized cell lines (e.g. murine BV2 microglia cells [45] and human HMO6 microglia cells [46]) to primary mouse and human cells, iPSC-derived models, as well as numerous rodent models. All models have both derived models. Mouse models, although valuable, are unable to faithfully replicate human neuroinflammatory responses due to species-specific differences in immune system architecture and signalling pathways [47]. For instance, it has been demonstrated that mice exhibit different immune responses to humans following injection of one of the most commonly used experimental neuroinflammatory activators, the glycolipid lipopolysaccharide (LPS) [48]. Amongst other species-specific differences, mouse astrocytes, but not human astrocytes respond to LPS [49]. As microglia and astrocytes are highly responsive to their environment, removal of primary human and rodent cells from the brain and further in vitro culture may skew these cells towards a more pro-inflammatory state that can affect subsequent experimental procedures. Indeed, single-cell RNA sequencing of cultured primary mouse microglia revealed a less homeostatic and more activated phenotype compared to freshly isolated microglia, which the authors described as a ‘culture shock’ transcriptome [50]. Primary human ex vivo microglia and astrocytes, which are most often derived from post-mortem samples, are difficult to obtain and manipulate, thus limiting their widespread use in research. Immortalized (human) cell lines, although easy to maintain and abundantly available due to their unrestricted proliferative capacity, have genetic and functional abnormalities, thus do not closely recapitulate microglia and astrocyte behaviour [51, 52]. The utilization of iPSC-derived cells overcomes these limitations by providing a direct and consistent source of human microglia and astrocytes, enabling investigation of human-specific responses [43, 53]. Moreover, iPSC-derived microglia and astrocytes can be generated from patient-specific iPSC, offering a personalized approach to understanding disease pathology. Their ability to model genetic variability and susceptibility enhances the translational relevance of findings and holds promise for drug screening and precision medicine approaches [54]. Disadvantages of iPSC models, however, include their resembling of a ‘foetal’ rather than an ‘adult’ state [53], considerable genetic variability between different iPSC lines [55], and the relatively high cost of iPSC generation and maintenance [56].

In this review, we summarise and access how neuroinflammation has been studied in human iPSC-microglia and iPSC-astrocyte cultures of increasing culture complexity. We highlight and critically evaluate which stimuli have been used to initiate an inflammatory response and mention the commonly used methods of detection, thereby providing a comprehensive guide on how best to model neuroinflammation in human iPSC models.

## Neuroinflammation studied in monoculture models

### iPSC-microglia monocultures

Multiple protocols are available for the differentiation of iPSC-microglia [57–63]. These protocols mimic microglia ontogeny by replicating in vivo pathways. Lineage-tracing studies in mice revealed that microglia progenitors are produced from mesodermal yolk sac macrophages, which migrate into the early brain before blood–brain barrier formation. Once inside the brain, these progenitors continue differentiating to a microglia phenotype [64]. Differentiation protocols attempt to recapitulate these events with a combination of growth factors and physical conditions that directs cells towards a mesodermal lineage and initiates early haematopoiesis. This results in the production of embryonic macrophage precursors which can be harvested and directed towards a specialized microglia phenotype. Microglia differentiation methods have been reviewed in more detail elsewhere [39], and some commonly used protocols are listed in Fig. 1A.

### Modelling neuroinflammation in microglia monocultures

iPSC-microglia monocultures have so far been the most studied iPSC model to investigate neuroinflammation (summarised in Table 1). Neuroinflammation is most commonly modelled by addition of a stimulant to the culture. Multiple stimulants are reported in the literature with the most common for iPSC-microglia being LPS. LPS is a component of Gram-negative bacterial cell walls and stimulates microglia through a series of interactions with several proteins, including the LPS binding protein, CD14, MD-2 and toll-like receptor 4 (TLR4) [65]. LPS addition results in a potent inflammatory response, with short stimulation paradigms of only 3–4 h shown to be sufficient in increasing gene transcription and secretion of multiple cytokines, such as IL-1β, IL-6, and IL-10, measured by RT-qPCR and enzyme-linked immunosorbent assay (ELISA) [66, 67]. However, much longer time courses are more commonly used ranging from 8 to 24 h, and prolonged LPS stimulation leads to an increase in further cytokines and chemokines [53, 58, 60, 68–73]. In addition to stimulation time, the concentration of LPS is important to consider. LPS concentrations are used in the range of 10 ng/mL to 1 µg/mL, with 100 ng/mL used most often. A brief comparison of 10 ng/mL and 100 ng/mL LPS stimulation for 6 h to unstimulated iPSC-microglia revealed that 10 ng/mL is sufficient to cause a significant increase in IL-1β and IL-6 gene transcription, as well as IL-6 protein secretion. In the same study, stimulation with 100 ng/mL LPS induced IL-1β gene transcription and IL-6, IL-1β and TNF protein secretion [66]. Between studies there is a huge variability in the cytokines that are reported to be upregulated by LPS, suggesting that experimental condition, e.g. duration and concentration of the stimulant, can have a huge effect on measuring neuroinflammatory findings. It would be advisable to include multiple stimulation timepoints in study designs, where possible, to ensure that the peak effect is captured. Another consideration when using LPS is that LPS derived from different *E. coli* strains are reported to have different biological effects, for instance on nitric oxide release [74].

**Table 1:** Stimulation procedures and outcomes for microglia monocultures

Stimulus	Stimulus concentration	Stimulus time	Method	Measure of inflammation	References
LPS	10 ng/mL	4 h	RT-qPCR	Increased *IL1B, IL6* Decreased *CX3CR1*, *TREM2*, *CD33*, *CSF1R*	[66]
			ELISA	Increased IL-6	[66]
	50 ng/mL	24 h	RT-qPCR	Increased *IL1B*, *CCL2*, *TNFAIP3*	[68]
	100 ng/mL	3 h	RT-qPCR	Increased *IL1B, IL10, NLRP3*	[67]
			Western Blot	Increased NLRP3	[67]
		4 h	RT-qPCR	Increased *IL1B* Decreased *CX3CR1, TREM2, CD33, CSF1R*	[66]
			ELISA	Increased IL-6, IL1B	[66]
		8 h	RT-qPCR	Increased *IL1B*	[69]
		18 h	RNA sequencing	Increased * IL1B*, *TNF*, *IL6*	[70]
			RT-qPCR	Increased * IL1B*, *IL6*	[70]
		24 h	Multiplexed ELISA	Increased TNF, IL-6, IL-8, IL-10, IL-1α, CCL2, CCL4, CXCL10, CCL17	[53]
			Multiplexed ELISA	Increased IL-1β, IL-6	[71]
			Profiler human cytokine kit	Increased TNF, Serpin E1, IL-8, IL-6, IL-1Rα, IL-1β, ICAM-1, CXCL10, CXCL1, CCL5, CCL2, CCL1	[58]
			Human magnetic Luminex assay	Increased IL-6, TNF, IL-10, CCL2, CCL3, CCL4, CCL5, CXCL1, CXCL2	[72]
			Cytometric bead array	Increased IL-6, MCP-1, IL-8, RANTES, GM-CSF, TNF	[60]
	1 µg/mL	24 h	RT-qPCR	Increased *IL6*	[73]
			ELISA	Increased IL-6	[73]
IFN-γ	10 ng/mL	24 h	Human magnetic Luminex assay	Measured 10 cytokines, no change detected	[72]
	20 ng/mL	24 h	Multiplexed ELISA	Increased TNF, IL-8, CCL2, CCL3, CCL4, CCL17	[53]
			Cytometric bead array	Increased IL-6, MCP-1, IL-8	[60]
LPS and IFN-γ	100 ng/mL LPS and 10 ng/mL IFN-γ	24 h	Human magnetic Luminex assay	Increased TNF, CCL2, CCL3, CCL4, RANTES	[72]
	100 ng/mL LPS and 20 ng/mL IFN-γ	24 h	RT-qPCR	Increased *TNF* and *IL6*	[62]
			RT-qPCR	Increased *TNF, IL6, IL1B*	[75]
			Cytokine antibody panel membrane	Increased MIPα/β, TNF, IL-6, IFN-γ, CXCL1, CXCL10	[62]
			Human cytokine array kit	Increased CCL2, MIPα, IL-6, IL-8, PA1-1, CXCL1, CXCL10	[75]
			Multiplexed ELISA	Increased IL-1β, TNF, IL-6	[71]
			Cytometric bead array	Increased IL-6, TNF, MCP-1, IL-8, RANTES, GM-CSF	[60]
		48 h	Cytometric bead array	Increased IL12p40, IL12p70, IL-6, IL-4, IL-10, IL-1RA, TARC, TNF, IFN-γ, IL-23	[76]
IL-1β	20 ng/mL	24 h	Multiplexed ELISA	Increased TNF, IL-8, CCL3, CCL4, CXCL10, CCL17	[53]
Amyloid-beta (Aβ_1–42_)	1 µM	24 h	RT-qPCR	Increased *TNF*, *IL6*, *IL1B*, *RELA*	[73]
Amyloid-beta fibrils (Aβ-F)	0.2 µM	24 h	Cytokine array	Measured 36 human chemokines and cytokines, no change detected	[79]
Amyloid-beta oligomers (AβO)	3 µM	24 h	RT-qPCR	Increased *TNF*, *IL6*, *IL1B*	[78]
α-Synuclein monomers	750 nM	6 h	ELISA	Increased IL-6, TNF	[66]
α-Synuclein oligomers	750 nM	6 h	ELISA	Increased IL-1β, caspase-1, IL-6, TNF	[66]
α-Synuclein fibrils (αSYN-F)	0.5 µM	24 h	Cytokine array	Measured 36 human chemokines and cytokines, no change detected	[79]

As LPS is considered not to be very physiologically relevant for many disease applications, cytokines, such as IFN-γ or IL-1β, can be alternatively used to induce neuroinflammation in iPSC-microglia either on their own [53, 60, 72], or in combination with LPS [60, 62, 71, 72, 75, 76]. The dual stimulation paradigm allows for the cytokine to prime the microglia, resulting in a heightened inflammatory response. This priming response also triggers canonical activation of the inflammasome, which results in increased caspase 1 production, which then cleaves cytokines IL-1β and IL-18 to their active forms, the latter of which stimulates IFN-γ production [77]. Abud and colleagues compared a 24-h stimulation of iPSC-microglia with 20 ng/mL IFN-γ, 20 ng/mL IL-1β, or 100 ng/mL LPS. Multiplex ELISA data for 10 key cytokines and chemokines revealed that LPS had the largest effect on protein production with all but CCL3 showing large significant increases. Stimulation with IFN-γ and IL-1β showed increases in 7–8 cytokines with no significant changes in the secretion of IL-6 and IL-10 with either stimulant, and no change in CXCL10 with IFN-γ stimulation. When used in combination, a stimulation of 100 ng/mL LPS and 20 ng/mL IFN-γ is often selected. The combined stimulus induced an increase in multiple cytokines and chemokines when compared to unstimulated iPSC-microglia, including TNF, IL-6, IL-8, IL-1β, CXCL1 and CXCL10, measured by RT-qPCR or protein arrays [60, 62, 71, 75].

Neuroinflammation can also be modelled by the introduction of disease-related aggregates, which has been done to model the neurodegenerative disorders AD (using amyloid-β) and PD (using α-synuclein). Addition of 3 µM oligomeric amyloid-beta (AβO) to iPSC-microglia for 24 h resulted in a significant increase in IL-1β, TNF, and IL-6 gene expression, measured by RT-qPCR. The authors compared AβO stimulation to the stimulation with 100 ng/mL LPS and showed that the LPS challenge increased all three cytokines to a much larger extent. However, both compounds triggered morphological changes in the iPSC-microglia towards an amoeboid phenotype [78]. Ihnatovych and colleagues found that a 24 h stimulation with 1 µM of the peptide Aβ_1-42_ increased iPSC-microglial gene expression of TNF, IL-6, IL-1β and the NF-κB subunit P65 (encoded by the gene *RELA*) [73]. Rostami and colleagues found dissimilar results when stimulating iPSC-microglia with 0.2 µM amyloid-beta fibrils (Aβ-F). After stimulation of iPSC-microglia with Aβ-F for 24 h, a cytokine array measuring 36 human chemokines and cytokines revealed no significant differences compared to unstimulated iPSC-microglia [79]. This might be due to a lower peptide concentration used, as well as a different species of  amyloid-β.

Rostami and colleagues also used the PD-related peptide α-synuclein to study a neuroinflammatory phenotype in iPSC-microglia. Stimulation with 0.5 µM α-synuclein fibrils (αSYN-F) led to no significant changes in 36 chemokines and cytokines measured via cytokine array [79]. Trudler and colleagues, however, found that both α-synuclein monomers and oligomers can induce an inflammatory phenotype in iPSC-microglia, measured by ELISA after 6 h. Stimulation with 700 nM of α-synuclein monomers induced a significant increase in IL-6 and TNF. The same concentration of α-synuclein oligomers induced a significant increase in IL-6, TNF, IL-1β, and caspase-1 [66]. These findings indicate that neuroinflammation triggered by neurodegeneration-related peptides can be studied in iPSC-microglia cultures, although more research is needed to better understand the variable results.

When modelling neuroinflammation in iPSC-microglia, the choice of stimulation paradigm is not the only important factor to consider. Autocrine and paracrine feedback mechanisms regulate cytokine release, therefore, the plating density of cells can significantly impact the final output measure. Although no data has been published for the effect of plating density on cytokine release in iPSC-microglia, it has been shown to have a large effect on iPSC-macrophages [80]. Stimulation was performed with either 100 ng/mL LPS alone or in combination with 10 ng/mL IFN-γ for 24 h. The results reveal density-associated effects with both stimulation paradigms, where densely plated cells secreted significantly less TNF, measured by ELISA [80]. These findings demonstrate the importance of keeping consistent plating densities when investigating neuroinflammation in vitro.

### iPSC-astrocyte monocultures

Many protocols have been developed in recent years for generating iPSC-astrocytes [81–86], see Fig. 1A. Unlike microglia, astrocytes have a neuroectodermal origin and are derived from radial glia in vivo [41]. Astrogenesis, which broadly follows neurogenesis in humans, is initiated following activation of the JAK-STAT canonical pathway. To differentiate iPSC-astrocytes in vitro, iPSC are differentiated into neural progenitor cells, followed by addition of a mixture of growth factors such as CNTF and FGF2, which aids the development of mature astrocytes [41].

### Modelling neuroinflammation in astrocyte monocultures

Not only iPSC-microglia, but also iPSC-astrocyte monocultures have been used to investigate neuroinflammation in vitro (summarised in Table 2). The most commonly used stimulus for triggering an inflammatory response in astrocytes in vitro is TNF. TNF, known to be secreted by microglia, was shown to play a key role in astrocyte activation in both human and murine models [9, 87]. However, it is clear that several important species differences exist following TNF stimulation, with human astrocytes displaying an elevated and more divergent cytokine response when compared to mouse astrocytes [87]. For the stimulation of iPSC-astrocytes, TNF has been used at concentrations of 10 ng/mL to 100 ng/mL and stimulation times vary widely between studies. Stimulation with 50 ng/mL TNF for just 1.5 h was sufficient to induce NF-κB nuclear translocation, measured by immunostaining [88], and increased IL-6 and IL-8 were detected after 5 h of TNF stimulation using flow cytometry [89]. However, most studies to date used longer TNF stimulation times of 24 h up to 7 days [88, 90–94]. In order to measure neuroinflammation in iPSC-astrocytes, cytokine and chemokine gene transcription and protein secretion have been studied most often. Two studies performed bulk RNA sequencing comparing unstimulated iPSC-astrocytes to those stimulated with 10 ng/mL TNF for 5 days [92] or 100 ng/mL for 7 days [90]. The results are largely overlapping, indicating that numerous cytokines and chemokines are upregulated after TNF stimulation. In addition, GFAP was used as a marker of astrocyte activation. GFAP intensity, measured via immunostaining, was shown to be increased after 5 days of stimulation with 10 ng/mL TNF [88]. However, in a second study, 7 days of stimulation with 50 ng/mL TNF did not affect GFAP, measured by western blot [93]. Furthermore, it was shown that TNF stimulation induced NF-κB nuclear translocation and phosphorylation [88, 90]. In summary, these data indicate that stimulating iPSC-astrocyte monocultures in vitro with TNF appears to induce a robust and reproducible inflammatory phenotype. However, more research is necessary to reconcile different observations in the literature regarding effects of TNF stimulation on GFAP levels. Moreover, when interpreting outcomes, it is essential to factor in published reservations regarding the accuracy of GFAP as an indicator of astrocyte reactivity, rather than just indicating the presence of astrocytes [10].

**Table 2:** Stimulation procedures and outcomes for astrocyte monocultures

Stimulus	Stimulus concentration	Stimulus time	Method	Measure of inflammation	References
TNF	10 ng/mL	24 h	RT-qPCR	Increased *IL8, IL1B, IFNG, TNF, IL2, IL4, IL6, IL10*	[88]
			Cytometric bead array	Increased IL-8, IL-1β, IFN-γ, TNF, IL-2, IL-4, IL-6, IL-10	[91]
		48 h	Cytometric bead array	Increased IL-8	[91]
		5 days	Bulk RNA sequencing	Increased *IL6*, *C3*, *CXCL10, CXCL11* amongst others	[92]
			Immunostaining	Increased GFAP intensity	[88]
			Multiplex bead-based immunoassays	Increased GM-CSF, IL-1β	[94]
	30 ng/mL	7 days	Western Blot	Increased NF-κB phosphorylation	[90]
	50 ng/mL	1.5 h	Immunostaining	Increased NF-κB nuclear translocation	[88]
		5 h	Flow cytometry	Increased IL-8 and IL-6	[89]
		24 h	Cytometric bead array	Increased IL-8	[91]
		48 h	Cytometric bead array	Increased IL-8	[91]
		48 h	RT-qPCR	Increased *CCL5*, *CXCL8*	[93]
		7 days	Western blot	No change in GFAP expression	[93]
			ELISA	Increased IL-6	[93]
	100 ng/mL	7 days	Bulk RNA sequencing	Increased *IL8*, *C3, CXCL10* and *CXCL11* amongst others	[90]
			Western Blot	Increased NF-κB phosphorylation	[90]
IL-1β	10 ng/mL	5 h	Bulk RNA sequencing	Enrichment for inflammation-related GO terms	[89]
		5 h	Flow cytometry	Increased IL-6 and IL-8	[89]
		24 h	Cytometric bead array	Increased IL-8	[91]
		48 h	Cytometric bead array	Increased IL-8	[91]
		48 h	RT-qPCR	Increased *CCL5*, *CXCL8*	[93]
		5 days	Multiplex bead-based immunoassays	Increased IL-6, GM-CSF, TNF, IL-23, IFN-β, IFN-α	[94]
		7 days	Western Blot	Increased GFAP expression	[93]
			ELISA	Increased IL-6	[93]
	100 ng/mL	7 days	Bulk RNA sequencing	Increased *IL8*, *C3, CXCL10* and *CXCL11* amongst others	[90]
			Western Blot	No change in NF-κB phosphorylation	[90]
TNF and IL-1β	10 ng/mL TNF and 10 ng/mL IL-1β	1 h	RT-qPCR	Decreased *GFAP*	[96]
			Western blot	Decreased GFAP	[96]
			Immunostaining	Increased NF-κB nuclear translocation	[96]
		5 days	Multiplex bead-based immunoassays	Increased GM-CSF, TNF, IL-1α, IL-6, IL-10, IL-12, IL-23, IFN-β, IFN-α	[94]
		7 days	RT-qPCR	Increased *CCL5, CXCL8, C3, LCN2*	[96]
			ELISA	Increased IL-6	[96]
		24 h	Multiplex bead-based immunoassays	Increased C3a, MCP-3, I-TAC, GRO-a, sICAM-1, GM-CSF, IL-1RA, MIG, RANTES, IL-6, MCP-2, MIP-1a	[98]
IL-1α, TNF, and C1q	3 ng/mL IL-1α, 30 ng/mL TNF, and 400 ng/mL C1q	24 h	Multiplex bead-based immunoassays	Increased C3a, MCP-3, I-TAC, GRO-a, sICAM-1, GM-CSF, MIG, RANTES, IL-6, MCP-2, MIP-1a	[98]
			Western blot	No change in GFAP	[98]
			RT-qPCR	Increased *TNF* and *IL-1β*, decreased *GFAP*	[97]
			Whole proteome analysis	Enrichment of GO-terms related to immune responses, cytokine-associated signalling, and recruitment of peripheral immune cells following treatment	[147]
Amyloid-beta fibrils (Aβ-F)	0.2 µM	24 h	Cytokine array	Measured 36 human chemokines and cytokines, no change detected	[79]
α-Synuclein fibrils (αSYN-F)	0.5 µM	24 h	Cytokine array	Measured 36 human chemokines and cytokines, no change detected	[79]

The second most commonly used stimulus when attempting to activate iPSC-astrocytes in vitro is IL-1β, which is known to be secreted by activated microglia [95]. IL-1β has been used at a concentration of 10 ng/mL or 100 ng/mL for 5 h up to 7 days to stimulate iPSC-astrocytes. Using a short incubation time of 5 h led to an increase in IL-6 and IL-8, measured by flow cytometry [89]. Longer incubation times of 24 h to 7 days could show an increase in IL-8, IL-6, GM-CSF, TNF, IL-23, IFN-β, IFN-α, CCL5 and CXCL8, as well as increased GFAP [91, 93, 94]. No activation of NF-κB was observed in IL-1β-treated astrocytes, which is dissimilar to the downstream effects of TNF stimulation [90]. Two studies performed bulk RNA sequencing on IL-1β-stimulated iPSC-astrocytes. Santos and colleagues used a stimulation of 10 ng/mL for 5 h and reported an enrichment for inflammation-related GO terms such as inflammatory response, immune response, chemokine activity, and cytokine activity in the IL-1β-stimulated iPSC-astrocytes [89]. Zhou and colleagues compared the effects of 7 days of stimulation with either 100 ng/mL TNF or 100 ng/mL IL-1β to unstimulated iPSC-astrocytes. They found that both upregulate pro-inflammatory genes, but by widely different magnitudes. Cross-comparison of genes significantly upregulated by IL-1β and TNF treatments revealed a large overlap, however treatment with TNF led to a much greater number of differentially expressed genes [90]. Contradicting this finding, two studies found IL-1β to be a more potent activator of iPSC-astrocytes than TNF [89, 94]. After 5 h of stimulation with either 50 ng/mL TNF or 10 ng/mL IL-1β, Santos and colleagues found the response to IL-1β stimulation to be higher, measured via flow cytometry of IL-6 and IL-8 [89]. Using a multiplex bead-based immunoassay, Perriot and colleagues showed that stimulation with 10 ng/mL IL-1β for 5 days increased IL-6, GM-CSF, TNF, IL-23, IFN-β and IFN-α in iPSC-astrocytes, compared to only GM-CSF and IL-1β in iPSC-astrocytes stimulated with 10 ng/mL TNF for 5 days [94]. Overall, these findings indicate that both TNF and IL-1β can individually induce a neuroinflammatory phenotype in iPSC-astrocytes, but suggest that the degree of activation is highly dependent on the experiment setup. Contradictory findings between studies when comparing iPSC-astrocyte responses to IL-1β and TNF demonstrate how further research must be undertaken before firm conclusions can be drawn.

Several studies to date have investigated the effect of the co-stimulation with both 10 ng/mL TNF and 10 ng/mL IL-1β on iPSC-astrocytes. Hyvärinen and colleagues detected NF-κB nuclear translocation, increased IL-6 secretion, as well as increased transcription of CCL5, CXCL8, C3 and LCN2 after co-stimulation, but also a decrease in GFAP gene expression [96]. Perriot and colleagues report that co-stimulation with TNF and IL-1β resulted in a huge synergistic effect, enhancing the production of both pro- and anti-inflammatory mediators. Compared with IL-1β stimulation only, co-stimulation with IL-1β and TNF induced a massive increase in the secretion of GM-CSF, IL-6, IL-10, IL-12, IL-23, IFN-β, and IFN-α [94].

A seminal study by Liddelow and colleagues demonstrated how activated primary mouse microglia secreted IL-1α, TNF, and C1q, which in turn lead to astrocyte activation, followed by neuronal death [9]. Since its publication, several groups have attempted to replicate these findings using human iPSC-astrocytes. Soubannier and colleagues found increased TNF and IL-1β gene expression following 24-h treatment with the cytokine cocktail [97]. However, this group also found decreased GFAP expression following the treatment, contrasting with findings of Liddelow and colleagues. Moreover, when Barbar and colleagues performed the same triple-stimulation as Soubannier and colleagues, but compared the resulting cytokine releases with stimulations with TNF and IL-1β, they observed no or incredibly minor differences in the cytokine release profiles between the two groups, and no change in GFAP protein levels [98]. These data suggest that TNF stimulation, which was shared between the two conditions, may be more important regarding human iPSC-astrocyte activation than IL-1α and C1q.

To date, neuroinflammation in the context of neurodegenerative diseases has been mostly studied in iPSC-microglia. Rostami and colleagues did not only investigate the effect of Aβ-F and αSYN-F on iPSC-microglia, but also iPSC-astrocytes. However, just like for the iPSC-microglia, stimulation of iPSC-astrocytes with Aβ-F and αSYN-F did not result in changes to any of the 36 measured chemokines and cytokines [79].

More studies are needed to draw conclusions on the neuroinflammatory responses of iPSC-astrocytes to disease-related stimuli. This would include comprehensively testing of all cytokines thought to be important for astrocyte activation both in combination and individually. Moreover, differences in the responses of mouse and human astrocytes when stimulating with certain immune stimuli [49, 87] highlight the importance of human models when studying inflammatory responses.

To conclude this section on monoculture models, thus far neuroinflammation has been studied more often in iPSC-microglia than iPSC-astrocytes, however both cell types have been demonstrated to respond to inflammatory stimuli in vitro. Different stimuli were used to activate glia cells, with LPS being the most commonly used for iPSC-microglia, and TNF and IL-1β for iPSC-astrocytes. Disease-relevant stimuli such as Aβ and α-synuclein can be used, however, so far they showed inconsistent inflammatory reactions when added to iPSC-microglia cultures and no effect on iPSC-astrocytes, indicating that more studies are needed to fully understand the neuroinflammatory response to disease-associated peptides. Following the addition of inflammatory stimuli, monocultured glia release a range of inflammatory mediators that can be measured using several well-established techniques. Factors to take into consideration when attempting to measure neuroinflammatory responses in monocultured glia include choice of stimuli, concentration, treatment duration, and cell plating density.

## Complex 2D iPSC culture models

### Introduction to complex models

The establishment of iPSC-derived monoculture systems in the past 15 years has led to advances in the study of neuroinflammatory processes. Analysing iPSC-derived microglia and astrocytes in monoculture allow for a higher level of detail in their characterization, which can be challenging to attain with more complex models. However, microglia, astrocytes, and neuronal cells interact with each other in the brain environment, both via physical contact and secreted factors [99]. This constant communication between brain cells is of paramount importance as it results in reciprocal changes to gene expression and cellular function. Excluding cell–cell communication from neuroinflammatory models may therefore lead to oversimplified or inappropriate conclusions [99]. Methods for generating iPSC cultures with a higher degree of complexity have been established to simulate a more physiological CNS environment. 2D co-culture systems can be generally divided into two categories: (1) culturing one population of cells using the conditioned medium derived from another population or, culturing the two populations of cells in the same vessel separated by a porous membrane. In both instances, the main aim is to allow secreted factors to be shared without physical contact. (2) Culturing more than one cell type in a dish with physical contact to each other.

### Modelling neuroinflammation using iPSC-derived conditioned medium

#### Microglia and neurons cultured with astrocyte-conditioned medium (ACM)

Two decades ago, scientists speculated that astrocytes might play an important regulatory role in microglial differentiation. Using primary mouse microglia, Schilling and colleagues demonstrated that microglia ramification is controlled by the astrocytic factors TGF-β, M-CSF, and GM-CSF. Cultured primary microglia were treated for 1 day with primary mouse ACM, which induced significant microglia ramification compared to non-conditioned medium. Moreover, microglia ramification was inhibited by neutralizing antibodies against TGF-β, M-CSF, and GM-CSF [100]. Zhang and colleagues compared the morphology of primary mouse microglia that were either treated with primary mouse ACM, cultured with primary mouse astrocytes but separated by a transwell, or cultured with direct contact to astrocytes. Microglia showed a more complex morphology when treated with ACM and cultured in transwells with astrocytes, compared to control medium. In co-culture these microglia, however, showed an even more complex morphology, indicating that astrocytes regulate microglia ramification through contact-dependent and -independent mechanisms [101]. Nowadays, many iPSC-microglia differentiation protocols use TGF-β, M-CSF, GM-CSF and further growth factors (including SCF and IL-34) to differentiate iPSC to a microglia phenotype [39]. However, to our knowledge no research has been published presenting a comparison between standard microglia media and the use of iPSC-derived ACM for iPSC-microglia culture. Further studies are needed to explore the potential beneficial effects of ACM on microglia cultures, as well as to investigate the effects of ACM from pro-inflammatory stimulated astrocytes on microglia phenotypes.

Hyvarinen and colleagues compared human embryonic stem cells (ESC)-derived neurons cultured in the presence of ACM from either control or reactive iPSC-astrocytes (treated with 10 ng/mL IL-1β and 10 ng/mL TNF for 7 days, then washed out and collected 48 h later). No increase in apoptosis or cytotoxicity was observed in ESC-derived neurons cultured with the reactive ACM. ESC-derived neurons were functionally supported when treated with both control and reactive ACM, which led the authors to speculate that the treatment of astrocytes with IL-1β/TNF might represent a neurosupportive rather than a neurotoxic stimulus [96]. However, these results are in contradiction to previous observations by Liddelow and colleagues, who found that reactive ACM from primary rodent astrocytes was highly neurotoxic and caused cell death of ESC-derived neurons within 24 h of culture. In this study, rodent astrocytes were stimulated with IL-1α (3 ng/mL), TNF (30 ng/mL) and C1q (400 ng/mL) [9]. The striking differences observed in the two studies could be explained by the different stimuli and concentrations used to trigger astrocyte reactivity, as well as differences in cell biology between mature rodent astrocytes and human iPSC-astrocytes [87].

#### Microglia cultured with neuronal (precursor)-conditioned medium (N(P)CM)

Banerjee and colleagues derived iPSC-microglia by supplementing media with GM-CSF and IL-34 or by exchanging that medium with an increasing gradient of neuronal precursor cell conditioned medium of up to 50%. The comparison between the two media conditions revealed increased gene expression of common microglia markers, including TMEM119, CX3CR1, and SALL1, in the NPCM-treated microglia, measured by RT-qPCR [69]. In line with these findings, Muffat and colleagues reported that iPSC-derived microglia exposed to stem cell-derived mature neuronal-secreted factors via a transwell setup showed a gene expression signature closer to foetal human microglia, when compared with iPSC-microglia cultured in defined media, measured by RNA sequencing [62]. This indicates that N(P)CM can contribute to the differentiation of iPSC towards a microglia phenotype by providing factors normally present in the CNS. To our knowledge, no study has yet focused on investigating the effects of N(P)CM of injured/dying neurons on potential iPSC-microglia inflammatory responses.

#### Neurons cultured with microglia-conditioned medium (MCM)

Recently the effect of microglia-conditioned medium on neuronal cultures has been explored in the context of the neurodegenerative disorder Huntington’s disease. O’Regan and colleagues stimulated ESC-microglia, derived from control or mutant *HTT* gene carrying human ESC, with 1 µg/mL of LPS and 10 ng/mL of IFN-γ. The stimulated MCM was added to the culture of isogenic ESC-derived striatal neurons for 5 days. Surprisingly, no differences in neuronal cell identity, cell viability, or DNA damage markers were observed after treatment with activated MCM, compared to control MCM [102]. Further research on the effect of MCM from microglia stimulated with commonly used pro-inflammatory activators, such as LPS, on iPSC-neurons is needed to better understand the effect of neuroinflammatory secreted factors on neuronal identity and viability.

In conclusion*,* various groups have started to use conditioned medium to explore the complex communication of CNS cells using iPSC cultures, however the use of conditioned medium remains uncommon. A huge disadvantage of using conditioned medium is that the medium composition is undefined and can vary between batches, as well as that it is unclear which factors contribute to the observed findings. However, the use of conditioned medium could potentially answer questions of non-physical cell–cell communication in a simple iPSC-derived monoculture model.

### Modelling neuroinflammation in iPSC co-culture models

A more recently developed and complex way to study interactions between brain cell populations in vitro involves using a culture system where cells are in physical contact. Over recent years, an increasing number of groups have established novel iPSC-derived CNS co-culture models.

#### iPSC-microglia and neuron co-culture

The first iPSC-derived microglia–neuron co-culture protocols were established in 2017 [44, 53]. In the healthy brain, microglia are proposed to be maintained in a homeostatic state by a crosstalk of the neuronal CD200 glycoprotein and microglial receptor CD200R, as well as interaction between microglial receptor CX3CR1 with the neuronal transmembrane chemokine fractalkine (CX3CL1) [103]. Abud and colleagues compared gene expression of iPSC-microglia in co-culture with rat hippocampal neurons to iPSC-microglia monocultures supplemented with the neuronal factors CD200 and CX3CL1 via RNA sequencing. They demonstrated that the direct contact of microglia with neurons suppressed pro-inflammatory signalling and maintained a microglia homeostatic transcriptional state, indicating that CD200 and CX3CL1 are not the only interactions important for microglia–neuron crosstalk, especially in the context of microglia homeostasis [53]. Haenseler and colleagues described a protocol to co-culture iPSC-cortical neurons with iPSC-microglia. Unstimulated iPSC-microglia in co-culture with neurons displayed resting ‘surveillance’ motility similar to in vivo microglia behaviour. When stimulated with 100 ng/mL of LPS, 5 h post-stimulation microglia showed reduced ramifications and an increased area-to-perimeter ratio, indicative of an amoeboid, activated morphology. Cytokine response upon activation with 100 ng/mL of LPS and 100 ng/mL of IFN-γ was compared between monocultured and co-cultured iPSC-microglia measured with a Luminex multiplex bead array assay. Co-cultured iPSC-microglia exhibited lower pro-inflammatory cytokine secretion in both basal and stimulated conditions, and in fact, their stimulated cytokine profile was broadly anti-inflammatory [44], indicating that co-culture with neurons induces a more ‘homeostatic’ state in iPSC-microglia, which might be more representative of in vivo microglia. More recently, Vahsen and colleagues described a co-culture of iPSC-microglia with iPSC motor neurons. Transcriptomic analysis demonstrated a microglial signature for the co-cultured iPSC-microglia, and they also showed increased ramifications, indicative of a homeostatic microglial identity. When stimulated with 100 ng/mL of LPS and 100 ng/mL of IFN-γ, after 18 h co-cultured iPSC-microglia revealed an amoeboid morphology and clustered together. Cytokine and chemokine secretion after the stimulation was measured using a membrane-based supernatant proteome array. In comparison with microglia monocultures, co-cultures showed a moderately attenuated secretion profile, with the downregulation of CHI3L1 and serpin E1 [104]. The results from both studies support the hypothesis that microglia acquire a less activated status when in direct contact with neurons [105].

#### iPSC-astrocytes and neuron co-culture

Multiple iPSC-astrocyte and neuron co-culture protocols have been established in recent years, however with little focus on neuroinflammation. Santos and colleagues explored the consequences of co-culture of neurons with activated astrocytes on the viability and dendritic length of the neurons. iPSC-derived astrocytes were treated with either IL-1β (10 ng/mL) or control vehicle for 24 h and co-cultured with neurons for 48 h after the removal of the cytokine. They observed a large decrease in the survival rate and reduction in dendritic length of the neurons when co-cultured with activated astrocytes, in comparison to astrocytes treated with vehicle, demonstrating that IL-1β-stimulated astrocytes have a negative impact on the maturation and survival of neurons [89]. A similar study, using a microfluidic platform to culture iPSC-astrocytes and iPSC-neurons, gave contradictory results. The microfluidic device contained separate chambers for neurons and astrocytes allowing cell-to-cell interactions within microtunnels, where astrocyte processes connected with neuronal axons. Hyvärinen and colleagues explored the effect of reactive astrocytes using IL-1β (10 ng/mL) and TNF (10 ng/mL) on number and density of neuronal axons. Co-culture of reactive astrocytes with neurons led to increased axonal density compared to co-culture with control astrocytes. The authors, therefore, hypothesized that reactive astrocytes might display a neurosupportive function towards axonal growth [96]. The discrepancy between the studies might be explained by a difference in the activation strategy and suggests the need for further studies on neuroinflammatory responses to different cues in co-culture models.

#### Triple cultures of microglia, neurons, and astrocytes

iPSC-derived triple-culture systems have recently been developed to investigate complex cell–cell interactions in vitro*.* Ryan and colleagues established an iPSC-derived triple culture of microglia, astrocytes, and neurons for the study of cognitive disorders associated with infection from human immunodeficiency virus (HIV). They independently generated iPSC-neurons, iPSC-astrocytes, and iPSC-microglia, combined the three into a common culture, and performed experiments 14 days later. In this study, the authors compared the tri-culture system in the presence or absence of HIV infection and assessed the outcome of common antiviral therapies. Single-cell RNA-sequencing performed on the tri-culture confirmed several findings previously documented using in vivo models, including enhanced inflammatory responses by all three cell types upon HIV infection. Microglia appeared to be the main culprit in driving the initial inflammatory response to HIV infection, as examination of inflammatory genes revealed the largest change in this cell type compared to astrocytes and neurons [106]. HIV is a virus that infects only human cells, limiting the use of animal models for studying this infection, and therefore the iPSC-derived triple culture system offers a great tool for the study of HIV infection in the brain. Guttikonda and colleagues developed an iPSC-derived triple culture primarily for the study of secreted complement C3, as C3 has been reported to be increased under inflammatory conditions and to be implicated in neurodegenerative disorders such as Alzheimer’s disease [107]. Upon stimulation with 1 µg/mL LPS for 72 h, increased C3 levels, measured by ELISA, were observed in co-cultures of iPSC-microglia and iPSC-neurons, and to an even greater extend in triple cultures of iPSC-microglia, iPSC-neurons, and iPSC-astrocytes, suggesting a potentiation of C3 secretion via cellular crosstalk of microglia and astrocytes. Furthermore, LPS stimulation increased secretion of the cytokines IL-6, TNF, IL-1β, IFN-γ, GM-CSF, and IL-10, measured by ELISA, in microglia–neuron co-cultures and tri-cultures, but not in astrocyte–neuron co-cultures and neuronal monocultures [59]. Bassil and colleagues developed a high-throughput iPSC-derived triple culture to model Alzheimer’s disease pathology by adding Aβ_1-42_ oligomers to the culture. In comparison to a neuron–astrocyte co-culture, the presence of microglia in a triple culture resulted in decreased neuronal death and increased Aβ plaque formation, suggesting that the Aβ plaque formation may be neuroprotective. When a pro-inflammatory stimulus (50 ng/mL IFN-y, 100 ng/mL IL-1β, 50 ng/mL LPS) and Aβ_1-42_ oligomers were added to the triple culture system, microglial–plaque association was increased, but the neuroprotective effect of microglia was lost. The authors hypothesize that microglial activation in response to Aβ may be beneficial in plaque compaction and neural protection, but over-activation could counteract these benefits through toxic microglial activities such as cytokine secretion [108].

In summary, over recent years, several groups have established iPSC-derived co-culture systems, either with two or three cell types, in order to achieve more physiological culture conditions, as well as to model complex cell–cell interactions. These human-derived cultures have the potential to validate research findings generated by monocultures or animal models. Compared to more complex neural organoids, co-cultures have the advantage of being easily accessible for experimental manipulation and live-cell imaging. However, the absence of neuronal sub-types, oligodendrocytes, vasculature, and 3D tissue organization are a limitation of the model. Overall, iPSC co-culture models are a versatile, yet not too complex, system for the study of neuroinflammation. Future studies will show how this model contributes to understanding cell–cell communication between the major cell types of the CNS.

### Complex 3D iPSC culture models

#### Neural organoid models

Neural organoids offer the investigation of neuroinflammation in an organ-like structure containing several cell types and extracellular matrix, while being accessible for functional analysis and drug interventions. Due to the increase in complexity, culture time and cost, to date very few research groups have used organoid models to validate findings from iPSC monocultures. Recently, guidelines for the nomenclature of three-dimensional cellular models of the CNS have been defined, recommending that these are termed ‘neural organoids’ [109]. Neural organoids recapitulate (parts of) the developmental process of the brain that leads to the generation of the brain’s unique 3D arrangement, establishing specific and unique substructures of the brain [110]. Two different types of methodologies are used to generate neural organoids: unguided and guided methods. Unguided methods rely on spontaneous morphogenesis of stem cell aggregates, whereas guided organoid methods require external patterning factors for the differentiation towards desired lineages. Further details about methodologies are reviewed elsewhere [111].

Most neural organoids recapitulate the diversity of cells originating from a neuroectodermal lineage, including radial glia, interneurons, astrocytes, and oligodendrocyte precursor cells [112]. Astrocytes appear after radial glia and neuronal cell types and mature over time. In order to study mature astrocyte functions in neural organoids, prolonged cultures well over 100 days are needed [113, 114]. Most protocols lack cells of non-neuronal origin, including mesodermal-derived vascular cells and microglia [115]. Incorporating microglia is recommended, as microglia play crucial roles in neural development and diseases [19].

Distinct techniques are used to include microglia in neural organoids. Firstly, microglia can be separately differentiated from iPSC and subsequently added to the culture medium, where they adhere to the surface of neural organoids and migrate into the interior [53, 62, 116, 117]. This approach allows the use of defined numbers of microglia, however it also means that neural organoid and microglia differentiations have to be performed in parallel. Secondly, neuronal progenitor cells have the capacity to self-assemble into 3D neural organoids, and neuronal progenitor cells and microglia precursor cells can be combined to generate uniform and cell-type ratio-controlled neural organoids [118]. Finally, it has been demonstrated that microglia can arise alongside neural cell types in minimally patterned organoids, which avoid directing differentiation towards a single germ layer [119–122]. No microglia precursors need to be added in these protocols, however the timing of appearance of microglia varies between protocols and microglia numbers are heterogeneous between individual organoids. This induces high variability into an already complex system and potentially leads to variable results between similar samples, for instance when measuring cytokine concentrations in the supernatant. A summary of organoid models can be found in Fig. 1D. A major focus in recent years has been to improve culture conditions, for example by improving nutrient supply into the inner core of the organoids, as well as the development of region-specific neural organoid models. These approaches can help to improve reproducibility and robustness of organoid models for future studies. Compared to iPSC-monoculture, as well as co-culture, where it is easier to control for cell numbers and ratios, studying microglia and astrocytes in organoids can lead to high variability within groups, so that relatively high sample sizes should be considered.

### Modelling neuroinflammation using neural organoids

Neuroinflammation has been studied in cerebral organoids stimulated with 100 ng/mL LPS for 24 h and 72 h. Ormel and colleagues used both ELISA and RT-qPCR to demonstrate increased levels of the pro-inflammatory cytokines IL-6 and TNF, but not the anti-inflammatory cytokine IL-10, after LPS exposure. Both astrocytes and microglia were present in the studied organoids, and the increased cytokine levels are likely to be induced by the interplay between both cell types, however this has not been dissected [119]. When treated with a combined stimulus of 100 ng/mL LPS for 24 h, followed by 2 mM ATP for 30 min, microglia incorporated into ‘tubular organoids’, which also contained astrocytes, changed towards an amoeboid-like morphology, suggestive of activation. These tubular organoids secreted higher levels of IL-1β, IL-18, and TNF, measured by ELISA, and showed NLRP3 inflammasome activation, quantified by colocalization of NLRP3 and ASC via proximity ligation assay. The authors repeated the experiments with a second stimulus, 100 nM DAMGO, a synthetic μ-opioid receptor agonist, for 24 h. In rat experiments, where 1 µM DAMGO was delivered to the nucleus accumbens core via microdialysis, a significant increase in IL-1α, IL-1β and IL-6 was measured using flow cytometry [123]. Here, DAMGO induced a similar inflammatory phenotype to LPS/ATP stimulation in the tubular organoids, indicated by a change in microglia morphology, increased secretion of IL-1β, IL-18, and TNF, and NLRP3 inflammasome activation. Interestingly, the authors found that TNF levels were increased after LPS/ATP stimulation in tubular organoids but not in 2D iPSC-derived microglia monocultures, which could be explained by crosstalk of the different cell types (including astrocytes) present in the tubular organoids model [124].

Neuronal damage has been modelled in neural organoids using needles and focal laser injury. When pierced with a 25-gauge needle, microglia near the injury side were found to adopt a more amoeboid morphology, suggestive of activation [53]. After focal laser injury, proximal microglia were observed to react within minutes by extending a single long process towards the injury centre, contacting the damaged zone. They then rapidly migrated their cell bodies to surround the damaged area, while microglia distant from the injury site remained immobile [62]. No study to date has investigated neuroinflammatory mediators after neural damage in neural organoids.

Neural organoids have also been used to investigate neuroinflammation in the context of disease. Zika virus (ZIKV) infection was modelled in microglia-containing neural organoids. After virus exposure, microglia adapted an ameboid morphology, suggestive of activation. Expression of IL-6, IL-1β, and TNF was examined by qRT-PCR and was significantly increased in the ZIKV-exposed organoids. Furthermore, the virus exposure had an effect on astrocytes as the authors found a significant increase in GFAP + cells in the ZIKV-exposed organoids [118]. Alzheimer’s disease-related neuroinflammation was modelled by Song and colleagues in microglia-containing brain region-specific organoids. Stimulation of microglia-containing dorsal cortical organoids with Aβ_1-42_ oligomers for 72 h led to a significant increase in TNF, but not IL-6 gene expression measured by RT-qPCR. Furthermore, Aβ_1-42_ oligomer treatment induced an increase in reactive oxygen species production in microglia-containing ventral cortical organoids [125].

Until recently, specifically astrocyte inflammatory responses have not been investigated in neural organoids. A recent study utilized bioengineered neural organoids that included a method of selectively initiating astrocyte reactivity through a genetically encoded chemogenetic tool. This chemogenetic astrocyte activation elicited a dynamic inflammatory reaction, which was detected by RNA sequencing. A Kyoto Encyclopedia of Genes and Genomes (KEGG) analyses identified TNF signalling, IL-17 signalling, MAPK signalling, Kaposi sarcoma-associated herpesvirus infection, and NF-κB signalling as the top five significantly upregulated pathways. This tool allowed for the direct activation of astrocytes in neural organoids and offers an opportunity for further studies of astrocyte reactivity in a 3D culture system [126].

To summarize, recently researchers started to use 3D neural organoids to study neuroinflammation, however, to date there has only been a few studies, which mostly focussed on microglia changes after stimulation. Similar to 2D cultures, LPS is a commonly used stimulus. As mentioned above, human astrocytes have been reported to not respond to LPS stimulation [49], however within neural organoids, microglia stimulated by LPS can release factors, such as TNF and IL-1β [127], that can activate astrocytes. Therefore, the recorded inflammation in neural organoids is likely due to the communication of microglia and astrocytes, which resembles a model of the human brain cell–cell interaction. More detailed studies are needed to break down the communication between microglia and astrocytes in neuroinflammation, and organoid models could hold a useful tool to do that. Additionally, in these complex systems the effect of neuronal damage on inflammatory responses can be exploited, thereby providing a more physiological induction of inflammation than the addition of external factors to the culture media. Before deciding to use neural organoids for a research study, it should be considered to start investigating the research question using iPSC-derived monoculture or co-culture models. Neural organoids can be a great tool to validate findings from less complex models, as well as to interrogate cell–cell interactions.

## Xenotransplantation

### Microglia and astrocyte xenotransplantation models

In vitro*,* iPSC models have a limited variety of brain cell types and extracellular matrix, which are important for regulating microglia and astrocyte functions. Microglia are particularly plastic cells, and at a transcriptional level their gene expression changes dramatically when isolated from the brain and cultured in vitro [128]. This has led researchers to develop a method to culture iPSC-microglia in vivo, where iPSC-microglia precursors are transplanted into brains of live postnatal or adult mice, with or without prior chemical depletion of the mouse microglia and are allowed to establish and mature over a period of 2–4 months [53, 129–132]. The resulting xenotransplant iPSC-microglia, or ‘xenoMG’, morphologically and transcriptionally resemble freshly isolated adult human microglia [130, 131]. The success of these initial methods required immunodeficient mouse strains to avoid massive immune activation and astrocyte proliferation resulting from invasive brain surgery [53, 129, 131]. These mice lack B, T, and NK adaptive immune cells [133]. T cells are critical for microglia maturation during mouse brain development [134], and infiltrate the brain during chronic neurodegeneration where they are likely to interact with microglia [135], so this should be considered as a limitation of the model. More recently a new xenotransplantation model with non-immunodeficient mice was developed, which relies on trans-nasal injection of the iPSC-microglia precursors to minimize disruption of the blood–brain barrier and avoid glial or immune activation [136]. A further innovation in microglia xenotransplantation models has been to integrate iPSC-microglia into iPSC-derived forebrain organoids, and transplant these into the retro-splenial cortex of immunocompromised NOD/SCID mice [137]. This method has the advantage of allowing human microglia interactions with human neurons to be studied and manipulated in an authentic brain environment, as the iPSC-microglia were shown to remain within the organoid graft. However, it is not clear how faithfully the organoid recapitulates brain tissue organization, thus more detailed characterization of the model would be helpful.

Astrocyte maturation at least partly requires neuron synaptic activity and a 3D environment [138, 139], therefore iPSC-astrocytes are likely to achieve limited maturity in monoculture. Stem cell-derived astrocytes have also been successfully transplanted into the brains of mice. The first protocol for astrocyte xenotransplantation used human ESC- or iPSC-derived glial progenitor cells, which developed into both astrocytes and oligodendrocytes in the mouse brain [140, 141]. These were used to investigate the non-inflammatory phenotypes of astrocytes with schizophrenia- or Huntington’s disease-linked genetic mutations. More recently, pure iPSC-astrocyte progenitors injected into immunodeficient NOD-SCID mice were used to characterize the morphologies of human astrocytes in the presence of Alzheimer’s disease pathology [142]. Neuroinflammation was not explored in these astrocyte xenotransplantation studies, therefore there is large scope for future research with this iPSC model. A summary of xenotransplantation models for iPSC-microglia and iPSC-astrocytes can be found in Fig. 1E.

### Modelling microglial neuroinflammation using xenotransplantation

Neuroinflammation has been studied in xenoMG models using systemic LPS challenge, with 2–5 mg/kg injected intraperitoneally. This resulted in xenoMG adopting ‘amoeboid’ morphology, suggestive of activation, downregulation of the homeostatic marker P2RY12 and upregulation of the activation marker CD45, measured by immunostaining [130, 131]. In one xenoMG study, the transcriptomic signature of systemic LPS challenge (2 mg/kg LPS given as three intraperitoneal injections spaced 24 h apart) was directly compared to in vitro LPS treatment of iPSC-microglia (100 ng/mL for 24 h) and observed to have very limited overlap and fewer differentially expressed genes than in vitro LPS challenge of iPSC-microglia monoculture [131]. The difference between microglia responses to in vivo and in vitro LPS challenge may arise from the peripheral immune system acting as the signal intermediary in vivo, with very little LPS crossing the blood–brain barrier and directly activating microglia [143]. However, the different timescales used (84 h in vivo versus 24 h in vitro) could also have had a confounding effect. Brain injury has also been modelled in xenoMG models using focal laser damage, and 2-photon time-lapse imaging of microglia at the site of injury showed that xenoMG rapidly extend their processes toward the lesion, at the same speed as mouse microglia [131].

Additionally, neuroinflammatory responses to specific neurodegenerative disease-associated pathologies have been modelled by injecting protein oligomers into the brains of xenotransplanted mice [66, 129, 144]. Synthetic amyloid-β oligomers caused the majority of the xenoMG to adopt a ‘cytokine response’ transcriptional signature, with upregulation of IL-1β, IL-6, CCL2, and CCL4, whereas treatment with a ‘scrambled’ peptide used as a control resulted in a homeostatic transcriptional signature for most cells, measured by single-cell RNA sequencing [129]. Alzheimer’s disease brain tissue-derived soluble tau fractions caused xenoMG to accumulate phosphorylated tau protein, likely due to endocytic uptake [144]. Single-cell RNA sequencing revealed an inflammatory transcriptional signature, when compared with xenoMG from mice injected with healthy control brain extracts. Significantly upregulated genes were associated with immune response, the type-I interferon response pathway, and antigen processing and presentation [144]. Finally, synthetic α-synuclein oligomers, a stimulus associated with Lewy body diseases caused induction of inflammasome activity in xenoMG, measured by ICC for caspase-1 [66]. The direct acute injection of disease-associated protein oligomers into xenotransplantation models allows the response of iPSC-microglia to these oligomers to be dissected, however this is not useful as a model of neurodegenerative disease, since neurodegenerative diseases are chronic and multiregional in nature.

To summarize, microglia and astrocyte xenotransplantation models are currently the most complex iPSC models with the most authentic brain-like environment. For research questions where microglia/astrocyte ‘maturity’, and interactions with other brain cell types, extracellular matrix, or the peripheral immune system are critical, these models will prove invaluable. However, time, expense, difficulty of manipulation, and ethical considerations will most likely restrain use of these models. Recent research has shown that it is possible to model neuroinflammation in vivo using xenotransplanted iPSC-microglia, although this should be done with caution and bearing in mind the potential impact of species differences on immune responses, and the use of immunodeficient animals that have no adaptive immune system. It is likely that xenotransplanted iPSC-astrocytes will be similarly exploited for the study of neuroinflammation in future.

## Conclusion

Neuroinflammation plays a significant role in various brain disorders, including neurodegenerative conditions, and methods to study iPSC-derived microglia/astrocyte inflammatory responses are of great importance. To induce neuroinflammation in microglia, most studies to date used LPS at a concentration ranging between 10 ng/mL and 1 µg/mL, and measured changes between 3 and 24 h after stimulation. In astrocytes, TNF (ranging between 10 and 100 ng/mL, measured between 1.5 h and 7 days after stimulation) and IL-1β (10 ng/mL, measured between 5 h and 5 days) were most often used to induce a neuroinflammatory phenotype. Even though these stimuli are known to induce a pro-inflammatory phenotype and are by now quite well characterized, the cellular responses might not be relevant for every disease. Therefore, more research is needed to improve and validate stimulations with disease-relevant stimuli. Complex cultures, including co-culture models, neural organoids and xenotransplantation have the advantage that advanced methods, such as neuronal damage, can be used to induce a more physiological pro-inflammatory environment. Future studies will help to re-evaluate which stimuli to choose for neuroinflammatory research.

In order to measure inflammatory changes, most studies used cytokine secretion or gene transcription as an indicator of activation. As a best practise, measuring both, gene expression changes, but also the release of cytokines into the extracellular space, is recommended and will highlight different aspects of the neuroinflammatory cascade. Furthermore, as immune responses to stimuli evolve over time, measuring responses over a series of timepoints will often provide the most comprehensive picture of neuroinflammation [145, 146].

Here we discussed inflammatory findings in a range of iPSC models with increasing complexity: monoculture, the use of secreted factors, 2D co-culture with other iPSC-derived brain cell types, 3D neural organoids and xenotransplantation. The existence of multiple different protocols for the generation of iPSC models means that the choice of protocol will likely affect neuroinflammatory findings, which is something to consider when choosing a culture protocol. There are some disadvantages to increasing culture complexity. The cost of setting up and maintaining cultures increases, and the ease of manipulation, throughput, and reproducibility decreases. The main advantage is that improved complexity is anticipated to improve the maturity and ‘authenticity’ of microglia/astrocyte phenotypes, in addition to allowing specific interactions between cell types to be modelled, including microglia–neuron and astrocyte–neuron interactions. However, cell-type specific questions are difficult to address in models where the recorded neuroinflammatory response is likely due to an interplay of multiple cell types and their interactions. Research questions could first be investigated in iPSC monoculture models, and then further validated in increasingly more complex cultures. Evidence is limited for how the different iPSC-derived models affect microglia and astrocyte phenotypes, as direct comparisons are rare. However, Xu and colleagues performed a meta-analysis of single-cell transcriptomic data from multiple studies using iPSC-microglia monoculture, iPSC-microglia developed within cerebral organoids, and two xenoMG studies including their own, and combined these with datasets of primary human foetal and adult microglia. Clustering by principal component analysis showed that the foetal and adult primary microglia separated by the first principal component, implying that this represents differences in relative age or maturity. Interestingly, the monoculture and organoid-culture iPSC-microglia clustered closely with primary foetal microglia, whereas the xenoMG were closer to adult primary microglia [132]. This suggests that xenoMG are either more mature/aged or perhaps just more quiescent and ‘homeostatic’ in phenotype than other iPSC-microglia models. The underlying biological differences warrant further characterization.

Future studies comparing complex cell culture models of microglia and astrocytes would be highly beneficial to the field, particularly with regard to neuroinflammatory responses. Moving towards higher levels of iPSC model complexity is assumed to improve the ‘brain-like’ authenticity of inflammatory responses, however there is currently little evidence to support this assumption, given the scarcity of cross-model comparisons. More studies comparing the different iPSC-model systems are needed to aid future researchers in the design and interpretation of their iPSC experiments.

## Data Availability

No new data was generated for this article.

## Abbreviations

3D: Three-dimensional
Aβ-F: Amyloid-beta fibrils
AβO: Oligomeric amyloid-beta
αSYN-F: α-Synuclein fibrils
ACM: Astrocyte-conditioned medium
AD: Alzheimer’s disease
ALS: Amyotrophic lateral sclerosis
ATP: Adenosine triphosphate
CNS: Central nervous system
DAMPs: Damage-associated molecular patterns
ELISA: Enzyme-linked immunosorbent assay
iPSC: Induced pluripotent stem cells
LPS: Lipopolysaccharide
MCM: Microglia-conditioned medium
MS: Multiple sclerosis
NAMPs: Neurodegeneration-associated molecular patterns
NF-κB: Nuclear factor κ-light-chain-enhancer of activated B cells
NPCM: Neuronal precursor-conditioned medium
PAMPs: Pathogen-associated molecular patterns
PD: Parkinson’s disease
ROS: Reactive oxygen species
TLR: Toll-like receptor
xenoMG: Xenotransplant iPSC-microglia
ZIKV: Zika virus
